# Noncoding RNAs in Acute Myeloid Leukemia: From Key Regulators to Clinical Players

**DOI:** 10.6064/2012/925758

**Published:** 2012-09-18

**Authors:** Alessandro Fatica

**Affiliations:** Department of Biology and Biotechnology “Charles Darwin”, Sapienza University of Rome, 00185 Rome, Italy

## Abstract

Recent analyses have shown that human cells transcribe almost their entire genomes, implying the existence of a huge mass of ncRNAs. At the present, microRNAs are the most investigated regulative non-coding RNAs. Several studies have demonstrated that microRNAs play a crucial role in hematopoietic differentiation and hematological malignancies, including acute myeloid leukemia (AML). Aberrant expression of microRNAs has been associated with specific genetic abnormalities and clinical outcome of patients with AML. In addition, since microRNAs can function as either oncogenes or tumor suppressor genes, the potential of using these molecules as therapeutic targets opens up new opportunities in the future of AML therapy. The recent demonstration that other regulatory ncRNAs, in addition to microRNAs, are involved in hematopoietic cell differentiation and diseases, suggests that they may also have a biological relevance in AML. This paper will describe the role of ncRNAs in AML and discuss the expectations for the use of ncRNAs in diagnosis, prognosis, and therapy of AML.

## 1. Introduction

Traditionally biologists have concentrated their efforts on understanding the functions of coding genes. It may therefore be a little surprising that only a tiny fraction of the human genome encodes proteins, yet in contrast recent studies showed that the majority of our genome is transcribed into non-coding RNAs (ncRNAs) [1, 2]. NcRNAs include highly abundant and functionally important RNAs, such as ribosomal RNAs (rRNAs), transfer (tRNAs), small nuclear RNAs (snRNAs), and small nucleolar RNAs (snoRNAs). However, two classes of recently discovered ncRNAs, microRltNAs (miRNAs) and long ncRNAs (lncRNAs), appear to play a significant role in the regulation of gene expression programmes that occur in higher eukaryotes [3–5]. These ncRNAs may be involved in all levels of gene expression regulation within the cell and, eventually, they have also been implicated in many diseases, including cancers [6–9]. The role of these ncRNAs in normal and malignant myelopoiesis and their use as diagnostic and prognostic markers in acute myeloid leukemia (AML) are the subject of this paper. 

## 2. MicroRNAs in Normal and Malignant Myelopoiesis

At the present, microRNAs (miRNAs) are the most studied regulative non-coding RNAs. MiRNAs are 20–22 nt small RNAs that generally function by negatively control mRNA translation and stability via recognition of complementary target sites in the 3′UTR of mRNAs. The biogenesis of miRNAs, their regulation, and mode of action have been extensively covered in different reviews [10–13].

Hematopoiesis is a highly regulated process in which pluripotent hematopoietic stem cells (HSCs) give rise to all the blood lineages: the myeloid lineage, which comprise neutrophils, eosinophils, basophils, monocytes, macrophages, megakaryocytes, platelets, and erythrocytes; the lymphoid lineage, which includes T, B and natural killer (NK) cells [14, 15]. The development of myeloid cells depends on the activation of specific genetic programmes that are responsible for the reduction in cell proliferation, induction of apoptosis and the expression of lineage-specific myeloid genes [14–17]. Among master regulators of these programmes are transcription factors [16, 17]. miRNAs provide an additional level of control beyond the transcription factors. In particular, they play a crucial role in blood cell development by fine-tuning differentiation and adjusting the cell response to external stimuli [5, 18–22].

Acute myeloid leukemia (AML) is a heterogeneous hematopoietic malignancy in which immature myeloid progenitor cells accumulate in the bone marrow and eventually in blood and organs interfering with the production of normal blood cells [16, 17]. In AML, the accumulation of leukemic cells (also referred as blasts) arises from a failure of myeloid progenitors to mature, and therefore AML used to be classified in subtypes based on the stage at which normal differentiation is blocked in the leukemic blasts [16]. This failure is characterized by genetic and epigenetic alterations in progenitor cells that alter the expression or function of key transcription factors [17]. Noteworthy, cells derived from different AML subtypes can be induced to differentiate by specific agents into cells that resemble normal counterparts. In particular, acute promyelocytic leukemia (APL) represents a powerful *in vitro* model system to study granulopoiesis [16, 23, 24]. APL is characterized by chromosomal translocations involving the retinoic acid aeceptor  *α*  (RAR*α*) gene resulting in clonal expansion of hematopoietic precursors blocked at the promyelocitic stage of differentiation [25]. The treatment of APL cells with all transretinoic acid (ATRA) overcome, this block and induces granulocytic differentiation [23]. First hints of the involvement of miRNAs in myeloid differentiation came from *in vitro* differentiation studies using APL cells [26]. The first miRNA found to play a critical role in APL differentiation was miR-223 [26]. miR-223 is preferentially expressed in myeloid cells [27] and is induced by ATRA treatment of APL cells through the transcription factors CCAAT/enhancer binding protein *α* (C/EBP*α*) and PU.1 [26, 28–30]. These proteins are key players in myelopoiesis as they regulate many myeloid-cell-specific genes [17]. Conversely, miR-223 expression is negatively regulated by NFI-A and E2F1 [26, 30]. NFI-A represses granulopoiesis and favours the development of erythrocytes [31], while E2F1, a critical regulator of the cell cycle, interferes with myeloid differentiation and promotes proliferation of myeloid progenitors [31, 32]. Notably, these two genes are posttranscriptionally regulated by miR-223, thus generating a negative feedback loop [26, 30]. Low levels of miR-223 have been reported in AML patients with the t(8; 21) chromosome translocation, which is responsible for the production of the fusion protein AML1-ETO (also known as RUNX1-ETO) and it has been shown that the AML1-ETO protein inhibits miR-223 expression through its binding to the promoter region and the recruitment of chromatin remodelling enzymes [33]. Importantly, treatment of cells carrying the AML1-ETO translocation with hypomethylating agents, AML1/ETO inhibitors, or ectopic expression of miR-223 was able to arrest proliferation and stimulate myeloid differentiation [26, 31, 33]. Altogether these data indicated that the deregulation of miR-223 might contribute to the differentiation block underlying myeloid leukemia pathogenesis.

Consistently with the data obtained in malignant myelopoiesis, in normal myeloid differentiation of human cord blood CD34^+^ hematopoietic progenitor cells (HPSs), miR-223 levels increased during granulocytic differentiation and decreased during erythroid maturation [34]. The decline of miR-223 is a critical event for the expansion of erythroblast cells. This is mediated, at least in part, by the translational repression of NFIA and LMO2 by miR-223 [31, 34].

A miR-223 knock-out mouse has been produced [35]. The presence of miR-223 is dispensable for granulocyte cell fate specification and its absence produced an increase of granulocyte progenitors and altered granulocyte immunological function. Interestingly, expansion of granulocyte progenitors has been also reported in conditional knock-out mice for C/EBP*α*  [36]. As C/EBP*α*  inhibits cell-cycle progression by interfering with E2F1 activity [37], these data suggest that downregulation of E2F1 by miR-223 (see above) could be important for the granulocytic maturation process. 

The transcription factor Mef2c has been identified as a crucial target of miR-223 in mouse myeloid precursors [35] and, indeed, conditional knock-out of Mef2c within the miR-223 knock-out mouse rescued the proliferation abnormality but not the differentiation defect and the functionality of granulocytes [35], suggesting that additional miR-223 targets might be responsible for these phenotypes. 

Following miR-223, additional studies have identified different miRNAs activated by ATRA treatment in APL cell lines and primary APL blasts [19, 29, 38–43]. In particular, these studies identified miR15/miR-16, miR-26, miR-29, miR-107, miR-142, miR-342, and let7 between the miRNAs significantly induced, whereas miR-181b was found to be downregulated by ATRA. Many of these miRNAs target genes with important roles in cell proliferation, differentiation and tumorigenesis [19, 26, 38, 39, 43]. In particular, NFI-A, Bcl-2, and RAS transcripts were identified as relevant targets for miR-107, miR15/miR-16, and let7, respectively [29]. Notably, many of these miRNAs were also upregulated during granulocytopoiesis of normal CD34^+^ HPSs [40, 43] and they showed decreased expression levels in patients with AML [29, 33, 40, 41]. Ectopic expression for some of these miRNAs, such as miR-26a, miR-29a, miR-142-3p and miR-342, has been produced and, similarly to miR-223, it stimulated granulocytic differentiation of AML cells [38, 41, 43, 44]. Conversely, at least one miRNA, miR-100, was found to inhibit granulocyic/monocytic differentiation and to stimulate proliferation of AML cells [45]. This miRNA negatively regulated the expression of the phosphatase RBSP3 (also known as CTDSPL) leading to an increase in phosphorylated pRB levels and the activation of E2F1. Notably, RBSP3/CTDPSL gene also encodes for miR-26a, a miRNA that, similarly to its host gene, acts as a tumour suppressor in AML by controlling expression of positive cell cycle regulators [41].

AML cell lines have been also used to study the role of miRNAs in monocytic differentiation. Whereas ATRA treatment induces differentiation to morphologically and functionally mature granulocytes, 1,25-dihydroxyvitamin D3 (VitD3), phorbol myristate acetate (PMA), or 12-O-tetradecanoylphorbol-13-acetate (TPA) induces a monocyte/macrophage phenotype [46–48]. Microarray analysis identified different miRNAs modulated during monocytic differentiation of AML cell lines, in particular miR-424, miR-32, and miR-181 [49–51]. miR-424 is activated by the master myeloid transcription factor PU.1 and stimulated monocytic differentiation through translational repression of its target NFI-A [52]. In turn, the decrease in NFI-A levels is important for the activation of differentiation-specific genes such as M-CSF receptor (M-CSFr). In line with these data, both RNAi against NFI-A and ectopic expression of miR-424 enhanced monocytic differentiation [52, 53]. The interplay among these three components was found in AML cell lines as well as in human CD34^+^ HPCs induced to differentiate into monocyte by cytokines [52, 53]. Mir-32 is induced by VitD3 and negatively regulates the pro-apoptotic factor Bim [51]. Ectopic expression of miR-32 increased the differentiation response of AML cells to VitD3. In addition, miR-32 inhibition is sufficient to elevate Bim expression and sensitize AML cells to chemotherapy-induced apoptosis [51]. Conversely, miR-181 levels are decreased by VitD3 treatment [54]. This miRNA regulates the expression of the cell cycle regulator p27^kip1^, which is normally induced during monocytic differentiation, thus ectopic expression of miR-181 counteracts monocytopoiesis [54]. A relevant miRNA-regulated molecular circuitry in monocytic differentiation has been characterised in human CD34^+^ HPCs [55]. This study showed that the miR-17-92 cluster was downregulated during cytokine-induced monocytopoiesis of human CD34^+^ HPCs. This produced upregulation of their target gene AML1 and the transcriptional activation of its transcriptional target M-CSF receptor. In addition, AML1 was shown to bind the miR-17-92 cluster promoter and to inhibit its transcription, thus generating a regulatory loop [55]. Notably, miR-17-92 downregulation is also observed upon PMA- and TPA-induced differentiation of AML cells [56], and this cluster is also epigenetically silenced by PU.1 during monocytopoiesis in mouse [57], indicating the importance of miR-17-92 downregulation for myeloid differentiation. PU.1 also controls the expression of miR-146a, miR-342, miR-338, and miR-155 during monocytopoiesis of mouse myeloid progenitor cell lines [58]. Therefore, in addition to controlling the expression of myeloid coding genes, the master transcription factor PU.1 exerts its crucial function in monocytic differentiation by modulating the expression of several miRNAs. Ectopic expression of miR-146a in mouse HSCs was able to direct the selective differentiation of these cells into functional macrophages [58]. In addition, miR-146a knock-out in mice led to a myeloproliferative disorder and eventually myeloid malignancies [59, 60]. This phenotype has been attributed, at least in part, to NF-kB activation, a key event in inflammation-induced carcinogenesis. Thus, miR-146 has been proposed to function as a tumor suppressor in the myeloid compartment [59, 60].

The importance of miRNA in myeloid differentiation has been further proved by the deletion of the essential miRNA processing endoribonuclease Dicer in mouse myeloid progenitors [61]. This led to a block in monocytic differentiation and myeloid dysplasia, a cellular condition that may be considered as a pre-leukemic state. 

## 3. Other Noncoding RNAs in Normal and Malignant Myelopoiesis

There is a continually growing list of long non-coding RNAs (lncRNAs) that are associated with gene expression regulation and diseases [62]. However, very little is known about their precise function. LncRNAs generally indicate a RNA molecule longer than 200 nt without defined open reading frames, which can regulate gene expression through different molecular mechanisms [62]. To date, the number of human lncRNA genes is close to 9,000. However, only a few of them have been assigned a role in myelopoiesis. One of the first lncRNA to be identified in the hematopoietic system was EGO [63]. This lncRNA was identified in eosinophil differentiation of CD34^+^ HSCs where it stimulated differentiation and mature cell function by transcriptionally regulating eosinophil granule protein expression [62]. However, its mode of action has not yet been described. Subsequently, “antisense to PU.1 was discovered”, a lncRNA produced by an antisense transcript to the master hematopoietic transcriptional regulator PU.1 that negatively regulates PU.1 mRNA translation [64]. PU.1 levels are critical for normal hematopoietic development and suppression of leukemia, and it was suggested that PU.1 antisense lncRNA contributes to keep them from being too high in expressing cells [64]. Mechanism and function of “antisense to PU.1” lncRNA resemble the ones of microRNAs in hematopoietic differentiation. However, even if many antisense transcripts have been identified, this mechanism has not been described for the regulation of other genes. HOTAIRM1 was identified as one of the lncRNAs induced during ATRA-mediated granulocytic differentiation of APL cell lines [65]. This lncRNA is one of the numerous lncRNAs produced from the HOXA cluster and its knockdown attenuated the expression of different HOXA genes, which are important transcriptional regulation in normal and malignant hematopoiesis. HOTAIRM1 also modulated the levels of CD11b and CD18 transcripts, two granulocytic differentiation genes. However, these effects did not produce evident defects in differentiation [65]. Conversely to other two well-characterized lncRNAs produced from the HOXA cluster, HOTAIR and HOTTIP, which regulates chromatin-modifying complexes [61], the molecular mechanism of HATAIRM1 action is still not known. Last to be identified was “lincRNA erythroid prosurvival” (lincRNA-EPS). This lncRNA is one of the about 400 lncRNAs whose expression is modulated during erythropoiesis [66]. In particular, lincRNA-EPS promoted terminal differentiation and inhibited apoptosis of mature erythrocytes by inhibiting Picard expression, a pro-apoptotic gene, by a still not defined mechanism [66]. Like other studies on the identification of lncRNAs in hematopoiesis, also this work raises many additional questions for future investigation. First of all, it will be important to determine how these lncRNAs regulate gene expression and if, similarly to microRNAs, they might be utilized as diagnostic and prognostic markers in AML.

More recently, altered levels of another class of ncRNAs, the nucleolar snoRNAs, were found misregulated in AML cells [67]. In particular, increased levels of SNORD112–114 snoRNAs were found in primary AML blasts. The same study showed that ectopic expression of these snoRNAs promoted AML cell growth through Rb/p16 cell cycle regulation [67], indicating that, similarly to other ncRNAs, also snoRNAs may contribute to leukemogenesis.

## 4. MicroRNAs in the Diagnosis of Acute Myeloid Leukemia

A number of studies have demonstrated that miRNAs can be utilised as diagnostic marker in AML. More than 50% of AML is characterized by specific cytogenetic abnormalities [16, 68]. Many of these are chromosomal translocation involving hematopoietic transcription factors that produce oncogenic fusion proteins such as AML1–ETO (t(8; 21)), CBF*β*-MYH11 (core-binding factor-*β*-myosin heavy chain 11; inv16), fusion proteins involving MLL (mixed lineage leukaemia; t11q23), and PML-RAR*α*  (promyelocytic leukaemia-retinoic acid receptor-*α*; t(15; 17)). In the remaining AMLs without apparent translocations, refer to as cytogenetically normal AMLs (CN-AML), a number of relevant molecular abnormalities have been described, such as the internal tandem duplication (ITD) or mutation of FMS-like tyrosine kinase 3 (FLT3) gene, mutations in nucleophosmin (NPM1), C/EBP*α*, and in the Wilms' tumour genes. Importantly, prognosis of AML patients is strictly correlated to the detected cytogenetic and molecular abnormalities [68].

MicroRNAs have been shown to act as oncogenes or tumor suppressor genes [6]. Therein, improper miRNA expression may also play an important role in leukemogenesis [18, 20, 21, 69]. Different miRNA expression analyses have been performed by large-scale miRNA profiling methods and specific miRNA expression profiles have been associated with specific chromosomal translocations [70–73]. Among the deregulated miRNAs in AML, these studies identified miRNAs already known to be hematopoietic specific (e.g., miR-142-5p, miR-223, and miR-181) or reported to be highly expressed in other hematological malignancies and solid tumours (e.g., miR-221, miR-222, miR-17-92 cluster and miR-155) [6, 18, 20, 21, 69]. In addition, different miRNAs with known tumor suppressor activity were shown to be downregulated (e.g., let-7 and miR-34) [6]. However, the mechanism(s) underlying regulation of miRNA expression and miRNA function in AML has been described for only few miRNAs (Table 1).

Aberrant epigenetic regulation of miRNA genes as a consequence of molecular abnormalities has been described as one of the cause of miRNA misregulation. Fazi et al. observed a down-regulation of miR-223 in t(8; 21) AML samples [33] and showed that this was due to epigenetic silencing by the AML1-ETO oncoprotein (see above). The tumor suppressors let-7b and let-7c were also found down-regulated in AML with t(8; 21) and inv16 [74]. Conversely, miR-126/126* was specifically overexpressed in both t(8; 21) and inv(16) AMLs, the rearrangements resulting in the disruption of core binding factor (CBF) that, in turn, produces partial promoter demethylation of CpG island in which miR-126/126* is embedded [75]. miR-196b, located between homeobox A9 (HOXA9) and HOXA10 genes, has been found to be specifically overexpressed in AML patients with MLL rearrangement [74–78]. Increased miR-196b expression depends on MLL fusion proteins [78], and its overexpression in bone marrow hematopoietic progenitor cells led to an increase in proliferation and survival capacity [78]. Thus, these data indicate that miR-196b deregulation might play an important role in the myeloid differentiation block that occurs in AML. Notably, inhibition of miR-196b activity by antisense LNA (locked nucleic acids) oligonucleotides in BM cells transformed with the MLL-AF9 fusion gene decreased their proliferative capacity [78]. In AML patients with the rare t(2; 11) translocation a specific upregulation of miR-125b was identified [79]. Enforced expression of miR-125b in AML cell lines and CD34^+^ hematopoietic progenitor cells was able to inhibit myeloid differentiation and apoptosis, and conferred proliferation advantage to these cells [79–81]. In addition, increased expression of miR-125b in the bone marrow of mice was sufficient to induce a very aggressive and transplantable leukemia [81–83]. Therein, these data indicated that high levels of miR-125b might contribute to leukemogenesis. Another relevant player in AML with MLL rearrangement is the miR-29 family [44, 84]. In particular, members of this family were found downregulated in (11q23)/MLL and deleted in AML with loss of chromosome 7q, which encoded for the miR-29b-1 and miR-29a genes [44, 84]. Importantly, ectopic expression of miR-29b in AML cell lines and primary AML blasts induced apoptosis and inhibited proliferation [84]. Furthermore, inoculation of miR-29b mimics into xenograft tumors decreased tumor growth [84]. Several targets were identified for miR-29 function in AML, including MCL1, CDK6, IGFR, JAK2, and the DNA methyltransferases DNMT3a and DNMT3b [84]. Conversely, the mir-17-92 cluster was found to be particularly overexpressed in AMLs with MLL rearrangements [75]. Notably, this cluster was shown to inhibit monocytic differentiation of CD34^+^ hematopoietic progenitor cells through down-regulation of AML1 (see above).

Several miRNAs were found deregulated in APL, which is generally characterized by the t(15; 17) translocation [16]. The PML/RAR*α*  oncogenic fusion protein is directly responsible for the silencing of the tumor suppressors let-7c and miR-342. Notably, these two miRNAs were induced upon ATRA treatment of APL cell lines and primary t(15; 17) APL [38, 40, 44]; thus they might represent new markers in the therapeutic response in APL patients.

MiRNA expression has been also analysed in CN-AML patients. Specific miRNA signatures have been associated with mutations of the nucleophosmin (NPM1) and CEBPA genes [70–73]. AML with NPM1 mutations is characterized by high expression of the homeobox (HOX) genes and, notably, one of the down-regulated miRNAs identified in this study (miR-204) controls the protein levels of the two HOX genes HOXA10 and MEIS-1 [70]. The same study identified several upregulated miRNAs predicted to target CD34, a gene whose expression is frequently downmodulated in these leukemias. AMLs with CEBPA mutations are characterized by a more mature phenotype of malignant blasts and low expression of several homeobox genes [71]. Increased miR-181 levels characterized these leukemias [71], a miRNA known to be involved in lymphoid lineage differentiation and to inhibit monocytopoiesis [18, 54]. In contrast, in different studies performed on a high-risk subgroup with normal karyotype, decreased levels of miR-181 were identified [73]. In this case, the downregulation of the miR-181 family was suggested to contribute to an aggressive leukemia phenotype through mechanisms associated with the toll-like receptors and interleukin-1*β*  [73]. The causes of miRNA deregulation in CN-AML are probably connected to altered epigenetic and/or transcriptional regulation. Epigenetic silencing of miR-124a has been associated with EVI1 overexpression [85–87], a transcription factor that plays a critical role in prognosis of AML. Notably, miR-124a is a translational repressor of C/EBP*α*  [88], therein it might contribute to the differentiation block observed in AML by altering expression of the prodifferentiative transcription factor C/EBP*α*  [17]. Another epigenetically regulated miRNA is the oncosuppressor miR-34b [89]. This miRNA regulates cyclic AMP responsive element-binding protein (CREB), an oncogene involved in the pathogenesis of AML that is downregulated by aberrant hypermethylation [90]. Conversely, increased expression of the miR-17-92 cluster, which is associated with different tumors including AML, is due to decreased expression of the master myeloid transcription factor PU.1 that cause ineffective epigenetic silencing of the oncogenic miRNA cluster [57]. The transcription of the miR-17-92 cluster is directly activated by c-Myc [91]. C-Myc is frequently activated in AML and plays an important role in the induction of leukemogenesis [92]. The E2F transcription factors, a family of critical regulators of the cell cycle that are activated by the oncogene c-Myc, were among the first experimentally verified targets of the miR-17-92 cluster [91]. Moreover, E2F proteins can directly participate in the transcriptional activation of these miRNAs, establishing a negative feedback loop [91]. The tumor suppressor PTEN (phosphatase and tensin homolog), which is often mutated in human leukemia, the proapoptotic and regulator of leukocyte homeostasis Bim (also known as BCL2-like 11), and the cyclin-dependent kinase inhibitor CDKN1A (p21), a potent negative regulator of the G1-S checkpoint, have been identified as additional relevant targets of the oncogenic miR-17-92 cluster [91, 93, 94]. Another miRNA repressed by c-Myc in AML is miR-26a, which was found down-regulated in different AML subtypes [95]. Notably, miR-26a acts as a tumour suppressor in different types of cancer, such as lymphomas and liver, breast, and nasopharyngeal carcinomas by targeting positive cell cycle regulators [96–100]. Mutations in the protooncogene C-KIT, an important cell growth factor receptor, are frequent in AML and are generally associated with poor outcome [101]. miR-193a is a negative translational regulator of C-KIT mRNA [102]. In patients with AML this miRNA is downregulated because of inappropriate methylation of its promoter region and its expression was inversely correlated with C-KIT. Notably, ectopic expression of miR-193a in AML cells reduced cell growth and induced apoptosis and differentiation [102].

## 5. MicroRNAs in the Prognosis of Acute Myeloid Leukemia

The expression signature of some miRNAs has been found to be associated with clinical outcome and survival of patients with AML. Mutations in NPM1 and CEBPA genes are generally associated with favourable outcome while the FLT3-ITD has been linked to unfavourable outcome [103]. Increased expression levels of miR-181 were associated with favourable outcome in AML with both normal and abnormal karyotypes [75, 104, 105], and connected with CEBPA mutations [105]. In addition, it has been suggested that it may contribute to the partial erythroid differentiation reported in AML with CEBPA mutations [104, 105]. Another miRNA significantly associated with a prolonged overall survival is miR-212 [106]. However, the prognostic significance of miR-212 did not correlate with specific AML subtype [106]. Conversely, miR-155 was found significantly highly expressed in AML patients with FLT3-ITD and associated with poor prognosis [107, 108]. Notably, sustained expression of miR-155 in mouse hematopoietic stem cells produced a myeloproliferative disorder, indicating that this miRNA may play a relevant role in leukemogenesis [109]. The C/EBP*β*  transcription factor, which plays an important function in myelopoiesis [17], has been identified as a direct target of miR-155 [110]. However, the mechanism of miR-155 action in the myeloid lineages remains largely unknown. Different studies reported low expression of let-7b and miR-9 in patients with favourable cytogenetic translocations [76] while high expression of miR-191 and miR-199a adversely affected overall survival of newly diagnosed AML patients with predominantly intermediate- and poor-risk cytogenetic [77].

Recently, high expression of miR-3151 was discovered an independent prognostic marker for poor outcome in CN-AML [111]. MiR-3151 is encoded in an intron of the coding gene BAALC, which also associate with poor outcome when highly expressed in CN-AML [112]. The two genes impact on different outcome endpoints: high miR-3151 expression associated with shorter disease-free and overall survival, while high BAALC expression predicted failure of complete remission and shorter overall survival [111]. Thus, the combination of both markers may be useful to identify patients with the poorest outcome.

In conclusion, miRNA expression will serve as a diagnostic and prognostic marker that adds valuable information beyond the cytogenetic. 

## 6. Future Directions

Recent advances in the field demonstrated the feasibility of manipulating miRNA expression levels as a potential therapeutic strategy for cancer. Therein, it is very likely that we will see the development of new therapeutic options based on miRNAs into the clinic in the next future. Beyond the understanding of the function of specific miRNAs that constitute therapeutic targets, a great effort will be put in the development of standard procedures for rapid and sensitive detection of miRNAs. Indeed, as miRNAs were found to be stably present in human serum [113] they might become novel noninvasive biomarkers for pathological conditions, including cancer. The study of other ncRNAs in normal and malignant hematopoiesis is still in its infancy, but new important progresses are expected in this field. Thus, it is clearly predictable that other ncRNAs, in addition to miRNAs, will become crucial new players in the diagnosis, prognosis, therapeutic responses, and even therapy of human leukemias.

## Figures and Tables

**Table 1:** MicroRNAs with a define role in AML.

MicroRNA	Misregulation in AML	Relevant targets	Effects

miR-17-92	Overexpressed in AML with MLL rearrangements [75]	AML1 [55], BIM [93], PTEN [91], p21 [94]	Counteracts monocytopoiesis [55, 57]

miR-29b	Downregulated in AML with MLL rearrangements and deleted in AML with loss of chromosome 7q [44, 84]	MCL1 [84], CDK6 [84], IGFR [84], JAK2 [84], DNMT3a [84], DNMT3b [84]	Induces apoptosis and inhibits proliferation of AML cells [44]

miR-125b	Upregulated in t(2;11) AML [79]	LIN28A [83]	Inibits myeloid differentiation in AML cells and CD34^+^ progenitor cells [79, 80] Induces leukemia when overexpressed in mouse bone marrow [81, 82]

miR-146a	Downregulated in CN-AML [22]	NF-*κ*B [60]	miR-146a knock-out mice present myeloid malignancies [59, 60]

miR-155	Upregulated in FLT3-ITD AML [107, 108]	C/EBPb [110]	Produces a myeloproliferative disorder when overexpressed in mouse hematopoietic stem cells [109]

miR-181	Upregulated in AML with CEBPA mutations [71]	p27 [54]	Counteracts monocytopoiesis [54]

miR-196b	Overexpressed in AML with MLL rearrangements [74–78]	HOXB8 [114]	Induces proliferation and survival capacity when overexpressed in bone marrow progenitor cells [78]

miR-223	Dowmregulated in t(8;21) AML [33]	E2F1 [30], MEF2C [35], NFIA [26]	Induces granulocytc differentiation in AML cells [26, 30, 33] miR-223 knock-out mice present altered granulocyte immunological function [35]

miR-342	Downregulated in t(15;17) AML (APL) [40, 44]	n.d.	Induces granulocyitc differentiation in AML cells [38]
